# In Vitro and In Vivo Evaluation of a Cyclic LyP-1-Modified Nanosystem for Targeted Endostatin Delivery in a KYSE-30 Cell Xenograft Athymic Nude Mice Model

**DOI:** 10.3390/ph15030353

**Published:** 2022-03-14

**Authors:** Samson A. Adeyemi, Yahya E. Choonara

**Affiliations:** Wits Advanced Drug Delivery Platform Research Unit, Department of Pharmacy and Pharmacology, School of Therapeutic Sciences, Faculty of Health Sciences, University of the Witwatersrand, 7 York Road, Parktown, Johannesburg 2193, South Africa; samson.adeyemi@wits.ac.za

**Keywords:** targeted drug delivery, LyP-1 homing peptide, endostatin, nanoparticles, anti-angiogenic, necrosis

## Abstract

This work investigated the use of LyP-1 as a homing peptide for p32 receptor targeting on the surface of an endostatin (ENT)-loaded chitosan-grafted nanosystem intended for intracellular delivery of ENT and mitochondrial targeting in a squamous cell carcinoma (SCC) cell line (KYSE-30) model. The angiogenic factors for VEGF-C and MMP2 were assessed with in vivo evaluation of the nanosystem upon ENT release and tumor necrosis in nude mice with a KYSE-30 cell xenograft. The LyP-1-modified nanosystem revealed a three-fold decrease in proliferation at 1000 µg/mL compared with the control and facilitated receptor-mediated cellular uptake and internalization. In addition, targeting of the Lyp-1-functionalized nanosystem to mitochondrial and nuclear proteins in vitro and in vivo was achieved. Up to 60% inhibition of KYSE-30 cell migration was observed and the expressions of VEGF-C and MMP-2 as angiogenic markers were reduced 3- and 2-fold, respectively. A marked reduction in tumor mass was recorded (43.25%) with the control, a 41.36% decrease with the nanoparticles and a 61.01% reduction with the LyP-1-modified nanosystem following treatment in mice. The LyP-1-functionalized nanosystem targeted tumor lymphatics, instigated nuclear rupture and mitochondrial distortion, and decreased cell proliferation and migration with inhibition of VEGF-C and MMP2 expression.

## 1. Introduction

Angiogenesis remains a crucial process for the survival and growth of tumor cells (tumorigenesis) [1,2,3] and previous studies have shown that angiogenesis is linked to tumor metastasis, for example in squamous cell carcinoma (SCC) [4,5]. A study on the peptide endostatin (ENT) has demonstrated promising broad-spectrum results as an angiogenic inhibitor with relatively low toxicity [6]. However, the clinical success of ENT is short-lived as a result of a number of reported challenges that impede its application, such as the need for a more targeted approach to reach a chemotherapeutic effect, as well as overcoming its short in vivo systemic circulation half-life and instability [7].

In a previous study, CHT-g-PEI-PEG-NH2 nanoparticles were successfully synthesized for the effective delivery of ENT with a 4-fold increase in cytotoxicity against an oesophageal squamous cell carcinoma (OSCC) cell line (KYSE-30) [8,9]. Although significant cytotoxicity was achieved, the system was void on any specificity as a non-targeting approach. In order to improve the safety profile of the nanosystem, a more targeted approach is warranted.

Interestingly, various peptides have been used as ligands to design targeted nano-enabled chemotherapeutics to augment the delivery of anti-cancer molecules [10,11]. In this approach, targeted cells that are usually investigated in cancer nano-therapy include the endothelial cells of tumors [12], and more specifically receptors such as vascular endothelial growth factor (VEGFR), integrins (α4β3, α5β1), vascular cell adhesion molecule-1 (VCAM-1) and matrix metalloproteinase (MMPs). LyP-1 as a cyclic peptide is made up of nine amino acids (CGNKRTRGC) originated from human MDA-MB-435 breast cancer xenografts [7]. Both tumorous and endothelial cells share LyP-1 and it aggregates in the nucleus of both primary and metastatic tumors upon intravenous administration. LyP-1 binds directly to the lymphatic vessels in certain tumors and its distinctiveness as a targeting peptide rests squarely on its potential to induce apoptosis upon cell binding as well as its dual cellular uptake by tumor and lymphatic endothelial cells. Studies have shown that LyP-1 can also bind to the cell surface receptor mitochondrial protein (p32) [13].

Based on these previous studies and in order to improve the short-lived clinical outcomes of ENT as a potential angiogenic inhibitor for SCC as previously reported [14], the novelty of this study focused on the use of LyP-1 homing peptide as a p32 receptor targeting moiety on the surface of an ENT-loaded CHT-g-PEI-PEG nanosystem, for direct nuclei and mitochondrial targeting and intracellular delivery of ENT in a model SCC cell line (KYSE-30). Furthermore, VEGF-C (as an indicator of lymphatic angiogenesis) and MMP2 (a potent marker for cell invasion and metastasis in tumorigenesis) were also targeted employing the new LyP-1 functionalized ENT-loaded nanosystem.

The critical quality attributes related to the in vitro processing parameters as a result of Lyp-1 functionalization as well as the ex vivo and in vivo performance of the modified nanosystem were investigated to ensure desirable cellular targeting, internalization and co-localization. In addition, the targeting specificity, cytotoxicity and anti-proliferative effects of the modified nanosystem were evaluated using a KYSE-30 cell line simultaneously with an enzyme-linked immunosorbent assay (ELISA) to assess the angiogenic factors for VEGF-C and MMP2. In vivo assessment of the modified nanosystem was also undertaken to assess the release kinetics of ENT from the LyP-1-functionalized nanosystem in athymic nude mice with a KYSE-30 cell xenograft as the preclinical model.

## 2. Results and Discussion

### 2.1. Structure and Function Modification for the LyP-1 Functionalized Nanosystem Synthesized

**Figure 1:**
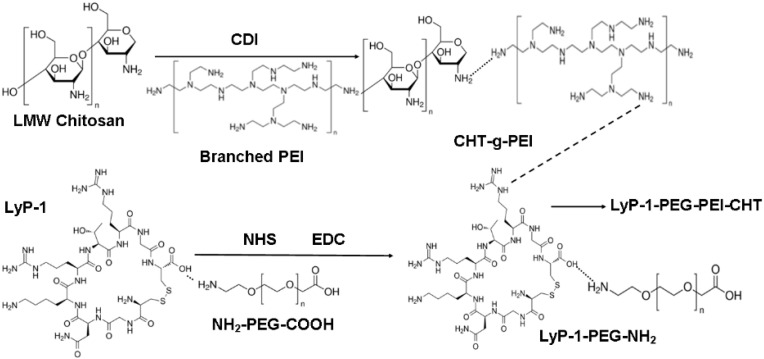
Reaction scheme for the synthesis of the LyP-1-functionalized nanosystem.

A multi-step approach was used to synthesize the CHT-g-PEI-PEG conjugates as the Lyp-1 substrate and core polymeric platform for the ENT-loaded nanosystem as per the reaction schematic in Figure 1. The CDI imidazoles facilitated coupling of the CHT and PEI amines (CHT-g-PEI conjugates) while the COOH moiety of NH2-PEG-COOH was linked to the amine of LyP-1 to form the LyP-1-PEG-NH2 conjugate. Acetamidomethyl (Acm) deprotection of the thiol of Cys2 and Cys10 using iodine reagent was employed to cyclize the attached LyP-1 peptide in order to enhance its stability [14]. The free amine of LyP-1-PEG-NH2 was then linked to the CHT-g-PEI amine as previously discussed. The ternary LyP-1-PEG-PEI-g-CHT conjugate formed was then loaded with ENT for further experiments.

**Figure 2:**
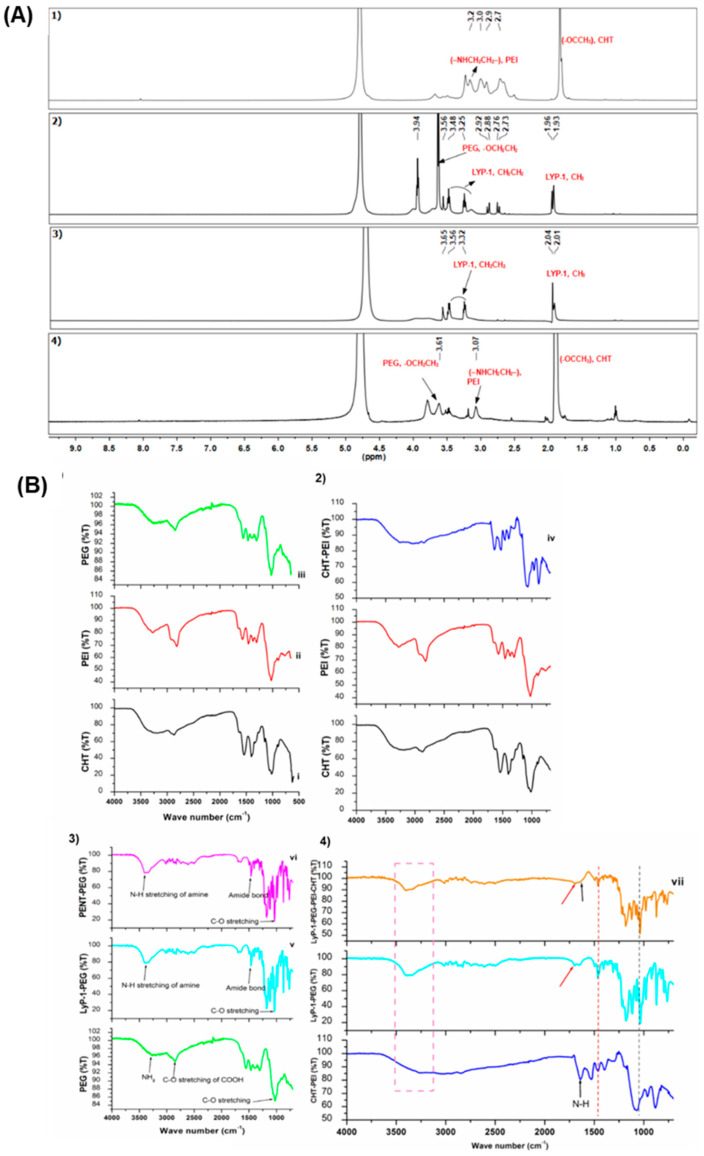
Structural and functional modification of LyP-1-functionalized nanosystem. (**A**) ^1^H NMR profile of the co-polymers in D_2_O:CH_3_COOD (5:1): (**1**) native chitosan-based conjugate, (**2**) linear LyP-1-conjugate, (**3**) cyclic LyP-1-conjugate, and (**4**) cyclic LyP-1-functionalized conjugate. (**B**) FTIR spectra of native and LyP-1 modified nanosystem. (**1**) Pure polymers including CHT, PEI and PEG, (**2**) spectra showing peaks for CHT, PEI and CHT-g-PEI nano-conjugate, (**3**) spectra of PEG and LyP-1-PEG-NH_2_ conjugates, and (**4**) spectra of CHT-PEI, LyP-1-PEG-NH_2_ and LyP-1-PEG-PEI-CHT nano-conjugates.

Figure 2A presents the NMR spectra of the conjugated constructs. Characteristic peaks of CHT, assigned at δ = 1.9 ppm, indicate the CH3^+^ of the nitro-acetyl group of chitosan (Figure 2A (1)) while peaks between δ = 2.5 and 3.2 ppm are attributed to the CH3 protons (–NHCH_2_CH_2_–) in PEI, confirming the successful grafting of CHT to PEI using CDI as a cross-linker. Proton NMR spectra for both the linear and cyclic LyP-1 peptides confirmed the cyclization of linear LyP-1 peptide to a more stable cyclic LyP-1 peptide. There is an upfield shift in the methylene signals for cyclic LyP-1 (3.25, 3.48) relative to the linear LyP-1 peptide (3.32, 3.56) (Figure 2A (2 and 3)). Interestingly, both spectra confirmed the conjugation of LyP-1 to NH2-PEG-COOH to yield LyP-1-PEG-NH2 conjugates. The repeating units of PEG appeared in the range 3.7–3.8 ppm in the 1H NMR spectrum of cyclic LyP-1-PEG-NH2 conjugate [6]. Upon the linking of CHT-g-PEI to LyP-1-PEG-NH2 conjugates, the appearance of the characteristic peak of PEG protons around 3.61 ppm confirmed the formation of the LyP-1 complex as shown in Figure 2A (4). Similarly, the characteristic peaks of both PEI and CHT were retained in the spectrum at 3.1 ppm and 1.9 ppm, respectively.

FTIR spectra and detailed absorption peaks of the polymers and conjugates are presented in Figure 2B (1); characteristic peaks of CHT, due to the C-O bend of the pyranose ring, was assigned at 886.69 cm^−1^ (Figure 2B (1(i))), while the signals at 854.83 cm^−1^ and 1465.07 cm^−1^ were assigned to the –NHCH_2_CH_2_ stretch in PEI (Figure 2B (1(ii))). Similarly, the peak at 841.33 cm^−1^ (from the -CH_2_CH_2_O-]n) represented the presence of PEG (Figure 2B (1(iii))). Absorption peaks at 3038 cm^−1^ and 3376 cm^−1^ indicated C-H and N-H stretching of the imidazole between the chitosan and PEI NH_2_ groups in the grafted conjugate (Figure 2B (2(iv))) [7]. Reduction in the frequencies of the characteristic peaks of both CHT at 882.85 cm^−1^ and PEI at 828.80 cm^−1^ confirmed the successful grafting of the two polymers. Of interest are the formations of the amide bonds in the LyP-1-PEG-NH2 conjugate. As seen in Figure 2B (3(v–vi)), the peak at 1697.46 cm^−1^ represented the C=O stretch absorption N-H bond I, 1463.54 and 1495.23 cm^−1^ were attributed to N-H bend and C-N stretch absorption in N-H bond II, while the triple peaks (1310.28, 1282.89 and 1261.99 cm^−1^) were assigned to the planar N-H bend and C-N stretch in N-H bond III. A peak at 3395.04 cm^−1^ indicated the N-H stretch from the NH_2_ bound to the PEG. Upon the conjugation of the LyP-1-functionlaized amino terminal PEG onto CHT-g-PEI, there seemed to be a small increase in the overall amine density. The absorption peaks of the free terminal amine shifted to a higher frequency of 3408.00 cm^−1^ from 3395.04 cm^−1^. Similarly, the frequencies of the amide bonds shifted slightly to 1697.54 cm^−1^ (amide I), 1463.55 cm^−1^ and 1496.61 cm^−1^ (amide II); and 1310.56, 1280.18 and 1253.22 cm^−1^ in amide III (Figure 2B (4(vii))).

#### In Vitro ENT Release from the LyP-1-Functionalized Nanoparticles

**Figure 3:**
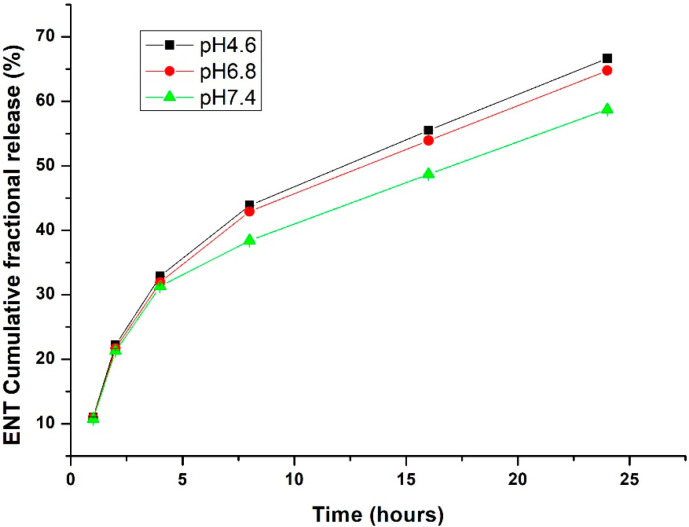
In vitro ENT release from the Lyp-1-functionalized ENT-loaded nanosystem. A relative increase in ENT was released from the LyP-1-functionalized nanosystem at acidic pH of 4.8 of the tumor microenvironment than at normal physiological pH of healthy cells.

A relationship exists between nanoparticles’ response to their environment and their intricate material composition. Nanomaterials can be tailored to release their payload at a predetermined rate based on material erosion, or diffusion of the captured payload through the nanoparticle matrix or pores [15]. Certain external stimuli, such as pH, can influence the release of their payload through alteration of their physicochemical properties. Interestingly, the tumor microenvironment exhibits a more acidic pH than the physiological pH of healthy cells [16]. Thus, nanoparticles can be tailor-made to withhold or release their payload in response to the pH of the cell’s micro- and macro-environments. Nanoparticle dissolution was tested at pH values of 4.6 (cell endolysosomal acidication simulation), 6.8 (tumor microenvironment) and 7.4 (physiological pH). As presented in Figure 3, LyP-1-functionalized nanoparticles release more ENT within the nanoparticle matrix at acidic pH values (i.e., 4.6 and 6.8) (*p* < 0.05). There is no significant difference in the release pattern of ENT between the functionalized and native nanoparticles. As such, functionalizing the nanoparticle surfaces does not influence ENT release but rather enhances the direct homing of the nanoparticles to their active sites within the cell. In this manner, sub-optimal release of ENT that often accounts for its high dosage was overcome with optimal release at the disease site with increased therapeutic effect. More importantly, the reduced ENT release at physiological pH will enhance the stability of the peptide-modified nanoparticles, with limited drug release in the blood circulation and at healthy tissues, and subsequent toxicity reduction [17].

### 2.2. Influence of the Physicochemical Properties of the ENT-Loaded LyP-1-Modified Nanosystem on Cellular Uptake, Internalization and Co-Localization

Often, the cell uptake of nanoparticles and their internalization, circulation, distribution and intra-cellular trafficking are influenced by their physicochemical properties [18]. A unique advantage of nanosystems in cancer therapeutics is their ability to pass through the porous tumor angiogenic endothelium and dysfunctional lymphatic drainage via passive transport into the tumor cells [15].

**Figure 4:**
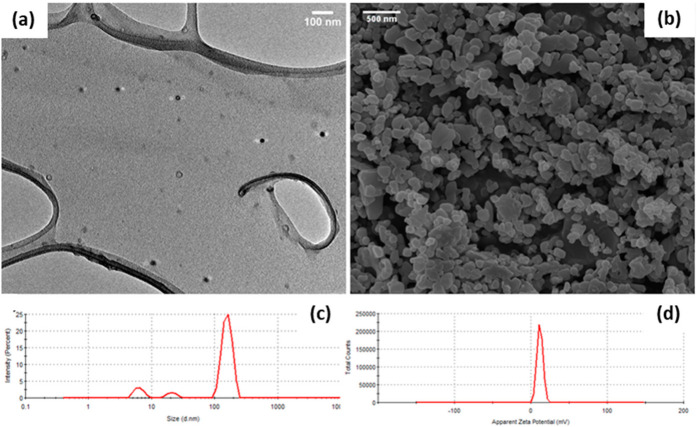
Size, shape and surface morphological characterization of LyP-1-functionalized ENT-loaded nanosystem. (**a**) TEM (60–120 kV) and (**b**) SEM (magnification: 25.00 kx) of the LyP-1-functionalized ENT-loaded nanosystem; (**c**) hydrodynamic particle size and (**d**) zeta potential (surface charge) of the LyP-1-functionalized ENT-loaded nanosystem.

The particle size and shape of nanosystems have been shown to influence their cellular uptake and internalization, circulation time, extravasation and interstitial diffusion within the cell [19]. Both TEM and SEM micrographs showed that spherical ENT-loaded LyP-1-modified nanoparticles were synthesized with an average particle size ranging between 30 and 200 nm (Figure 4a,b). SEM micrographs revealed that the surface of the nanoparticles appeared coarse due to peptide attachment. Meanwhile, the hydrodynamic size of the nanoparticles ranged between 100 and 106.45 nm as presented in Figure 4c. A polydispersity index of 0.46 was recorded for the nanoparticles, indicating their physical stability and relative homogeneity in size. Interestingly, lymphatic drainage of cancerous endothelial cells can allow for passive transport of molecules with sizes up to 150 nm (including SCC cells) [20]. More so, spherical nanoparticles have proven more efficient for cellular uptake and internalization into tumor microenvironments [15]. Thus, the Lyp-1-modified nanosystem synthesized had desirable size and shape parameters for effective internalization into a tumor microenvironment.

The surface charge (zeta potential) of the nanoparticles influenced their cellular binding, internalization and circulation time. Upon internalization, charged nanoparticles are targeted by phagocytes within the immune system through opsonization for destruction [21]. Based on previous studies [21], cationic nanoparticle surfaces preferentially bind to cells and internalize better when compared with neutral and anionic nanoparticle surfaces. Cationic nanoparticles are also preferentially engulfed by tumor cells in comparison to neutral particles [22]. Interestingly, the ENT-loaded LyP-1-modified nanoparticles possess an average zeta potential value of 17.3 mV as shown in Figure 4d. As such, the synthesized Lyp-1-modified nanosystem can bind more specifically to the anionic SCC cell surface [23] and be internalized through receptor-mediated endocytosis.

**Figure 5:**
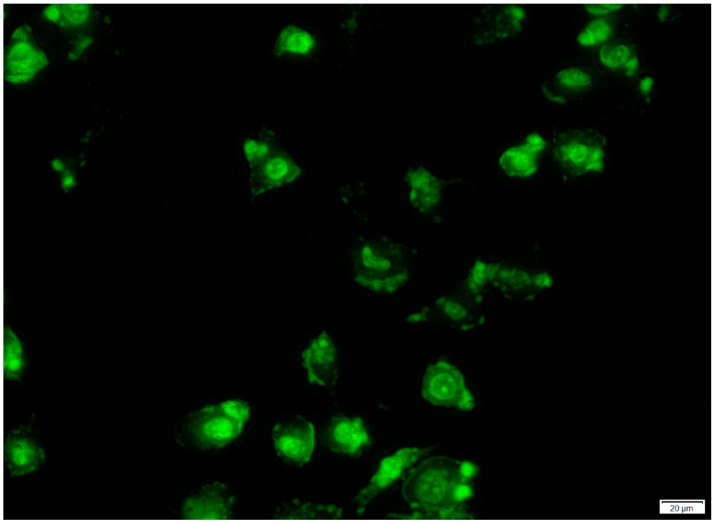
Confocal laser scanning microscopy (CLSM) micrograph showing cellular uptake and internalization of the LyP-1-enhanced ENT-loaded nanosystem. Nanoparticles were labelled with FITC.

**Figure 6:**
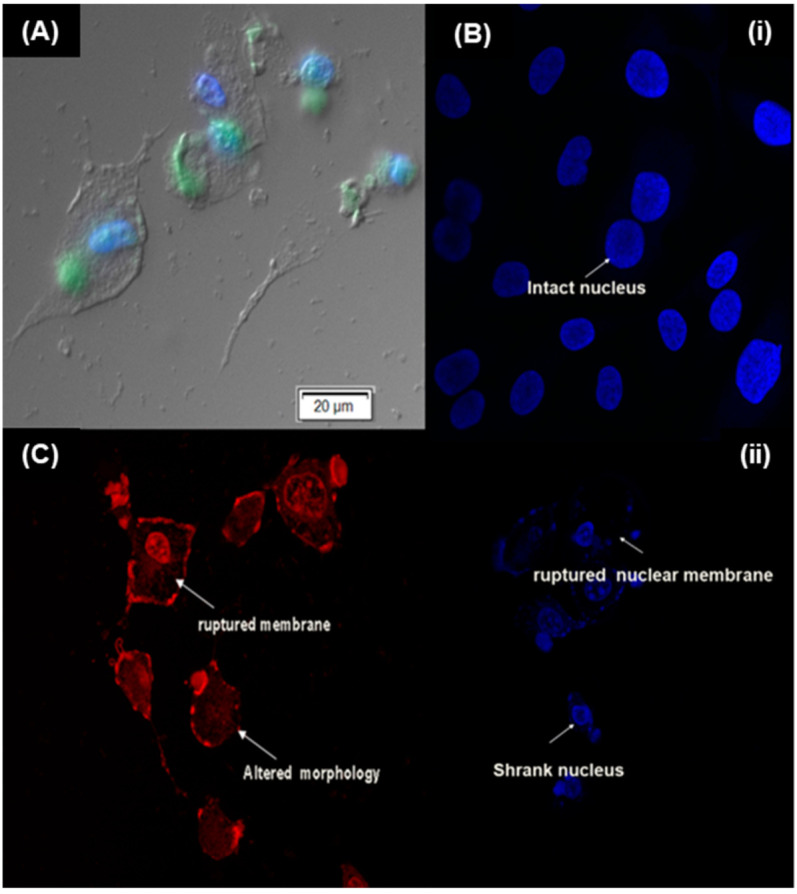
Effects of LyP-1-modified nanosystem on KYSE-30 nuclear and mitochondrial integrity. (**A**) Differential interference contrast (DIC) micrographs showing subcellular co-localization of the LyP-1-functionalized ENT-loaded nanosystem in the nuclei of KYSE-30 cells stained with DAPI. (**B**) CLSM images of the nucleus of KYSE-30 cells stained with DAPI: (**i**) untreated KYSE-30 cells; (**ii**) KYSE-30 cells pre-incubated with the LyP-1-functionalized ENT-loaded nanosystem. Depleted nuclear membrane showed signs of apoptosis and necrosis. (**C**) CLSM images of mitochondria stained with rhodamine 123 in live KYSE-30 cells. Altered-configuration of the mitochondrial morphology and disrupted mitochondrial membrane showed apoptosis [29].

**Figure 7:**
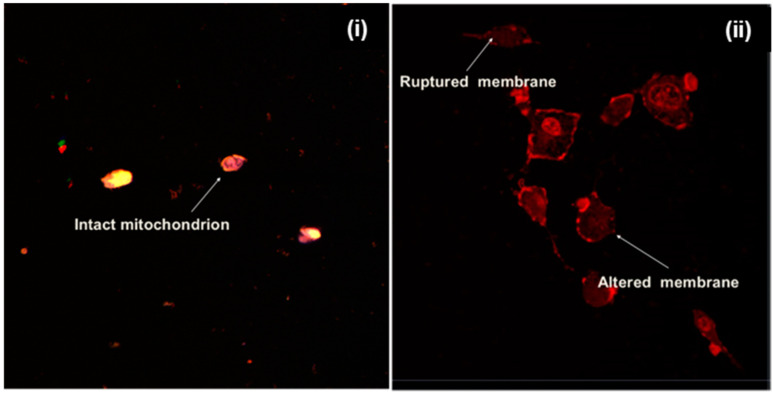
CLSM images of mitochondria stained with rhodamine 123 in live KYSE-30 cells. (**i**) Untreated KYSE-30 cells; (**ii**) KYSE-30 cells pre-incubated with LyP-1-functionalized ENT-loaded nanosystem. Changes in the configuration of the mitochondrial morphology and disruption of mitochondrial membrane showed apoptosis. Images were captured at 50 kx.

Beyond particle size and surface charge, targeting ligands [24] including peptides can be attached to nanoparticle surfaces to facilitate their direct binding and internalization into the cells [25]. Specific receptors that are over-expressed on certain tumor cells can serve as molecular signatures to facilitate the direct binding, internalization and intracellular delivery of peptide-functionalized nanosystems [26]. In this study, cyclic LyP-1 was bound to the surface of ENT-loaded nanoparticles. To account for LyP-1 stability and enhanced binding affinity, the Acm-thiol-protected Cys^2^ and Cys^10^ of the linear peptide were deprotected and cyclized using an iodine protection mechanism [14]. The binding affinity and stability of cyclic peptides has been shown to be greater than that of linear peptides [27]. Figure 5 shows green fluorescent particles luminescent within KYSE-30 cells with FITC-labelling of the LyP-1 modified nanoparticles. This was due to their interactions with the anionic surface of KYSE-30 cells. Similarly, FITC-labelled nanoparticles selectively co-localized into the nuclear and mitochondrial compartments within KYSE-30 cells (Figure 6). Differential interference contrast (DIC) images of the KYSE-30 cell nucleus (Figure 6A) and mitochondria (Figure 6C and Figure 7) show selective sub-cellular localization of LyP-1 modified nanoparticles within their compartments when stained with DAPI and rhodamine 123 fluorophores, respectively. Green fluorescent nanoparticles were condensed within the nucleus (blue) (Figure 6(Bi–Bii) and Figure 7) indicating the selective targeting of the nucleus for nuclear proteins such as nucleoline, which has been reported as a biomarker for endothelial cells in angiogenesis [28].

The combination of LyP-1 modification with cationic and spherical nanoparticles displayed selective binding affinity and internalization relative to other forms of surfaced-functionalized nanoparticles in cancer nanomedicines [29] of other forms that were different. These parameters also influence the intracellular trafficking, co-localization and therapeutic effects in biological systems [18].

### 2.3. Binding Specificity and Cytotoxic Effects of the ENT-Loaded LyP-1-Modified Nanosystem to KYSE-30 Cells

The morphological integrity of cell nuclei is a clear index of cell health [30]. A leaky and swollen nucleus with signs of early plasma membrane degradation and erosion is characteristic of necrotic nuclei, whereas apoptotic nuclei show DNA fragmentation, and nuclear and chromatin condensation with an intact plasma membrane [31]. In this study, the nuclei of KYSE-30 cells were labeled with DAPI to monitor the anti-angiogenic effects of the ENT-loaded LyP-1-modified nanosystem as shown in Figure 6B. As opposed to the nuclei of control cells, the nuclei of treated cells, i.e., with the ENT-loaded LyP-1-modified nanosystem after 24 h pre-incubation, showed nuclear plasma degradation, nuclear condensation and the release of nuclear materials into the cytosol. While the full understanding of the mechanisms of the anti-angiogenic effects of the ENT-loaded LyP-1-modified nanosystem has not yet been elucidated, it is clear that LyP-1 induced cell death by apoptosis [32]. Meanwhile, further investigation of key biochemical assays signaling compromise in nuclear material and apoptosis should be investigated to validate current findings.

The mitochondria are key organelles implicated in different types of cancers. Changes that affect the mitochondrial compartments, particularly DNA, can result in increased proliferation and avoidance of cell death pathways as observed in cancer cells [33]. Therefore, specific targeting of the mitochondria represents a strategic intervention in cancer nanomedicine since cancer cells utilize the mitochondria to increase disease progression. This is explained by the Warburg hypothesis that cancer cells exhibit a metabolic switch in their energy metabolism from oxidative phosphorylation to glycolysis and subsequently to lactic acid fermentation, a phenomenon Warburg attributed to defects in the mitochondria [34]. Meanwhile, current investigations showed that mitochondria depletion from cancer cells reduces tumor progression [35].

**Figure 8:**
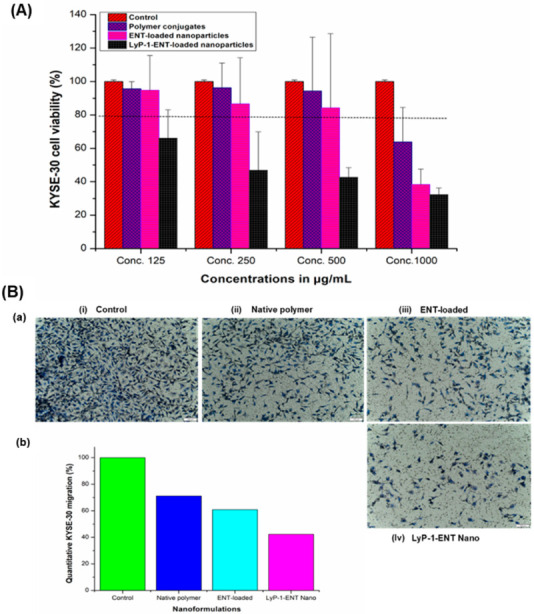
Cell proliferation and migration of KYSE-30 cells. (**A**) KYSE-30 cell viability after pre-incubation with nanosystems at varying concentrations for 24 h showing the proliferation effects of KYSE-30 cells. (**B**) Transwell migration assay of KYSE-30 cells pre-treated with native ENT, nanoparticles and the LyP-1-functionalized nanosystem. (**a**) Representative microscopic images of the transwell inserts of KYSE-30 cells after pre-incubation with different nanosystems: (**i**) control, (**ii**) native polymer conjugates, (**iii**) ENT-loaded nanoparticles, and (**iv**) LyP-1-functionalized ENT-loaded nanoparticles. The inserts show the macroscopic images of transwell inserts stained by trypan violet. Pores of the membranes could also be observed as the numerous small, round and dark colored dots in the picture. Images were taken at a magnification of 100 µm. (**b**) Histogram showing the quantification of the relative migration of KYSE-30 cells toward the chemo-attractant. Relative cell migration is expressed as the percentage of migrated cells with treatment compared to those without treatment (control). Results were computed from the average of triplicate samples with standard deviation.

Interestingly, LyP-1 homed specifically to mitochondrial protein p32 (receptor) that is over-expressed on the surface of cancer cells, including SCC [36]. A previous study has shown that rhodamine 123 selectively binds to the mitochondria of living cells [37]. Its cation at physiological pH binds preferentially to the abundant anions across the mitochondrial membrane. Hence, any external damage to the morphology of the mitochondria of living cells can be easily assessed. As presented in Figure 6C, the ENT-loaded LyP-1-modified nanoparticles affected a significant change in the mitochondrial configuration of KYSE-30 cells upon pre-incubation with the nanosystem. A ruptured mitochondrial membrane, distorted mitochondrial morphology and enlarged mitochondrial DNA, as seen in Figure 6C and Figure 7, are indices of apoptosis [33]. This in turn accounted for the reduced cell viability shown in Figure 8A. Results revealed that LyP-1, as a homing peptide, contributed to the enhanced in vitro cellular anti-angiogenic effects of the nanosystem.

### 2.4. Assessment of Anti-Proliferative Effects and Cell Migration

Proliferation and endothelial cell migration are the principal features of angiogenesis in tumor cells [3]. Increased cell survival and endothelial cell proliferation are the major channels by which cells that form new blood vessels are produced and maintained. At low-serum or low-growth-factor conditions, proliferation assays can be used to measure angiogenic activity, or to evaluate anti-angiogenic activity when the culture medium contains normal levels of serum and/or growth factors [38]. The Alamar blue assay was used to evaluate the cytotoxic effects of the ENT-loaded LyP-1 modified nanosystem on KYSE-30 cells [39,40]. As shown in Figure 8A, KYSE-30 cells were pre-incubated over 24 h with serum-free cell medium containing the pure conjugates, the nanoparticles and the LyP-1-modified nanosystems at varying concentrations. Among the examined concentrations, the polymer conjugates exhibited cell viability >80%, an index of superior cyto-compatibility in comparison to the control (untreated KYSE-30 cells) [41]. Interestingly, cells treated with the ENT-loaded LyP-1-modified nanoparticles showed reduced proliferation (by up to three-fold) at 1000 µg/mL compared to the control (*p* < 0.05). This was due to the targeting effects of LyP-1 in homing the ENT-loaded nanosystem specifically to the over-expressed nuclear and mitochondrial KYSE-30 receptor protein p32 that enhanced the ENT anti-angiogenic effects [42].

A major focus in the fabrication of anti-cancer nanomedicines is cell motility. Tumor invasion and angiogenesis depend on the migration of cells as a key determining factor [38]. The mechanism by which cancer cells spread necessitates their migration and invasion across the extracellular matrix (ECM), intravasation into the blood circulation or lymphatic vessels, attachment to distant sites after metastasis and a subsequent extravasation to form foci at another distant position [43]. As shown in Figure 8B (a), KYSE-30 cells treated with the ENT-loaded LyP-1-modified nanoparticles showed decreased migration of >60% when compared to the motility of the control cells. Cells pre-incubated with the native polymer conjugates showed a slight increase in migration than cells pre-exposed to the ENT-loaded nanoparticles (*p* < 0.05). This, however, is expected as ENT released from the nanoparticles induces apoptosis, thereby reducing the number of migrated cells across the trans-membrane pores in the Boyden chamber as shown in Figure 8B (b).

### 2.5. Anti-Angiogenic Measurement Using VEGF-C and MMP2 Assays

**Figure 9:**
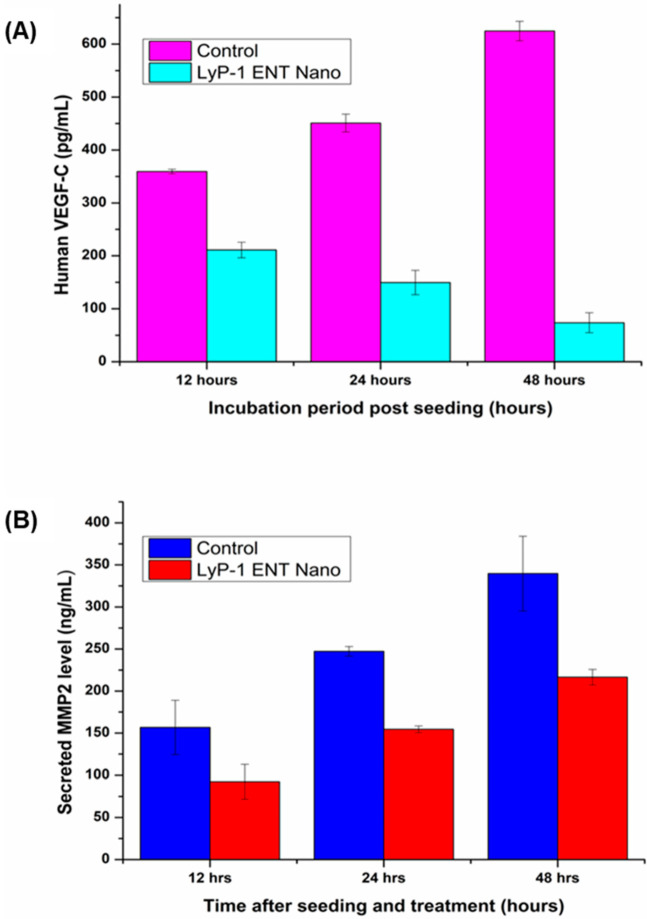
Anti-angiogenic assessment of functionalized LyP-1 nanoparticles. (**A**) VEGF-C expression in SCC KYSE-30 cells under cell culture conditions. KYSE-30 cells in culture conditions were washed with PBS and cultured in fresh medium without FBS as control. The LyP-1-functionalized ENT-loaded nanosystem was dissolved in free FBS culture media (2.5 mg/mL) as the treatment group. Culture supernatants were harvested at 12, 24 and 48 hours post-treatment by ELISA assay. Concentration of VEGF in supernatants was represented as pg/mL in duplicate. (**B**) Effects of ENT on MMP-2 production by KYSE-30 cells. KYSE-30 cells (80% confluent, in a 75 cm^2^ flask) were incubated with the LyP-1-functionalized ENT-loaded nanosystem for 48 h (each at 2.5 mg/mL). Untreated KYSE-30 cells were used as the control. Cell supernatants were harvested for MMP2 assay by ELISA. Samples were prepared in duplicate. Error bars indicate ± standard deviation.

ENT is known to reduce tumor growth by inhibiting angiogenesis [44]. Tumor cells enhance the angiogenic process by producing angiogenic factors, such as basic fibroblast growth factor (bFGF) and vascular endothelial growth factor (VEGF), in particular. Interestingly, ENT is a viable anti-angiogenic protein that blocks VEGF-C, a member of the VEGF family [45]. Similarly, ENT suppresses tumor and endothelial cellular invasion by binding to the active site of MMP2, thereby blocking its activation and catalytic activity [46]. VEGF is a key factor that mediates tumor angiogenesis associated with the development of tumor blood supply and the progression of cancer in response to hypoxia [47]. As shown in Figure 9A, the ENT-loaded LyP-1-modified nanoparticles reduced VEGF-C expression over the incubation period. While there was a progressive increase (1.74-fold increase) in the secretion of VEGF-C into the conditioned media by the control (untreated) KYSE-30 cells over 48 h, VEGF-C expression was reduced up to 2.9-fold in cells pre-incubated with the ENT-loaded LyP-1-modified nanosystem (*p* < 0.05). This phenomenon of reduction in VEGF-C expression is congruent with a previous study by Kim and co-workers [48], where ENT inhibited VEGF-mediated signaling through direct interaction with KDR/Flk-1 in vivo. Interestingly, a previous study has also shown that LyP-1 blocked lymphangiogenesis-related receptors, such as VEGF signaling proteins and nucleolin expressed on lymphatic vessels on primary tumors and metastatic lymph nodes [44]. Therefore, in this study, LyP-1 enhanced the anti-angiogenic effects of ENT by suppressing VEGF-C expression.

ECM degradation is a key factor linked to the invasion and metastasis of cancer cells and MMPs play a crucial function in this process [49]. MMP-2 facilitates the invasion and metastasis of cancer cells to the lymph nodes. As shown in Figure 9B, ENT inhibited the expression of MMP-2 relative to the control (untreated) KYSE-30 cells. The untreated cells continuously secreted MMP-2 into conditioned media as shown by the progressive elevation in MMP-2 levels over the 12–48 h incubation period. In relation to the control, cells treated with the ENT-loaded LyP-1-modified nanosystem did not follow the time-dependent increase in MMP-2 expression as observed in the control. This suppression in MMP-2 expression caused by ENT occurred 12 h post incubation with the ENT-loaded LyP-1-modified nanoparticles, where its expression was reduced by up to 1.7-fold compared to the control. Similarly, the inhibition of MMP-2 expression was maintained for up to 48 h and resulted in a 1.5-fold reduction for the ENT-loaded LyP-1-modified nanoparticles compared to the control (untreated). This result is in line with the findings of Kim and co-workers [46] who showed that ENT suppresses tumor and endothelial cell invasion by inhibiting the catalytic and activation domains of MMP2. A study has also shown that LyP-1-conjugated nanoparticles contributed immensely to the direct targeting of lymphatic metastatic tumors [14]. Thus, the anti-MMP2 suppression displayed by ENT could have been aggravated through a complementary role of LyP-1 peptide in enhancing the onsite targeting of ENT-loaded nanoparticles to enhance their efficacy.

Overall, these results demonstrate that both VEGF and MMP2 expression and secretion levels were reduced following pre-incubation with the ENT-loaded LyP-1-modified nanoparticles, suggesting that the LyP-1 modification described in this study could enhance the delivery of ENT for improved VEGF/MMP2-driven in vitro anti-angiogenic activity in SCC.

### 2.6. In Vivo Assessment of the ENT-Loaded LyP-1 Modified Nanosystem in a Xenograft

#### Effects on Tumor Volume and Mouse Weight

**Figure 10:**
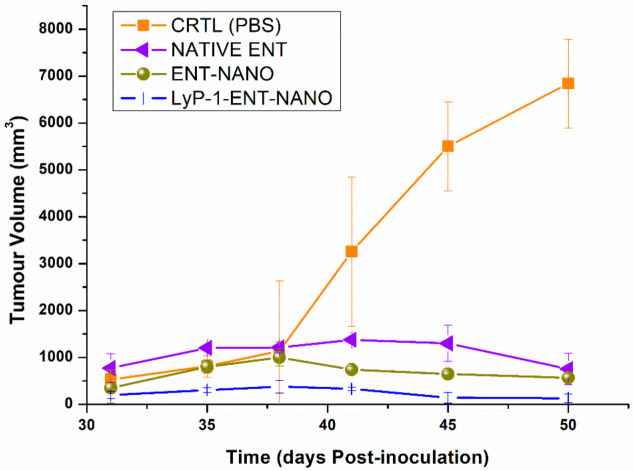
Therapeutic effects of the LyP-1-functionalized nanosystem treatment in KYSE-30 xenografts. Error bars indicate ± standard deviation.

Angiogenesis is over-expressed in tumor progression, tumorigenesis and metastasis and widely validated for its activation in SCC [50,51,52]. VEGF is an angiogenesis marker [53] that is upregulated in SCC as proven from surgical samples of patients managed by curative surgery [54]. In addition, it has been reported that enhanced microvessel density (MVD) is directly proportional to the progression of disease, tumor stage and its size, as well as the depth of tumor invasion in SCC [55]. As shown in Figure 10, mice within the experimental group displayed improved tumor size reduction. Interestingly, the ENT-loaded LyP-1-modified nanosystem revealed enhanced anti-tumor effects on tumor volume compared to the control (Figure 10). In summary, all tumor volumes were elevated up to 5505.54 mm^3^ in the control group and largely diminished within the treatment groups to 128.23 mm^3^ (97.67%) (*p* < 0.05) post-inoculation (31 days) and emergence of solid tumors. There was a decrease in tumor size from 1000.2 mm^3^ to 567.64 mm^3^ (43.25%) (native ENT group), 324.43 mm^3^ to 190.25 mm^3^ (41.36%) (ENT-loaded nanosystem) and 328.86 mm^3^ to 128.23 mm^3^ (61.01%) (ENT-loaded LyP-1-modified nanosystem) after treatment. Tumor volume in mice treated with ENT-loaded nanoparticles decreased up to 2-fold compared with native ENT. The Lyp-1-modified nanosystem therefore contributed significantly to the targeted delivery of ENT to enhance its anti-angiogenic activity. LyP-1 modification facilitated the direct and active transport of ENT for tumor volume reduction in the treated mice group (Figure 10).

**Figure 11:**
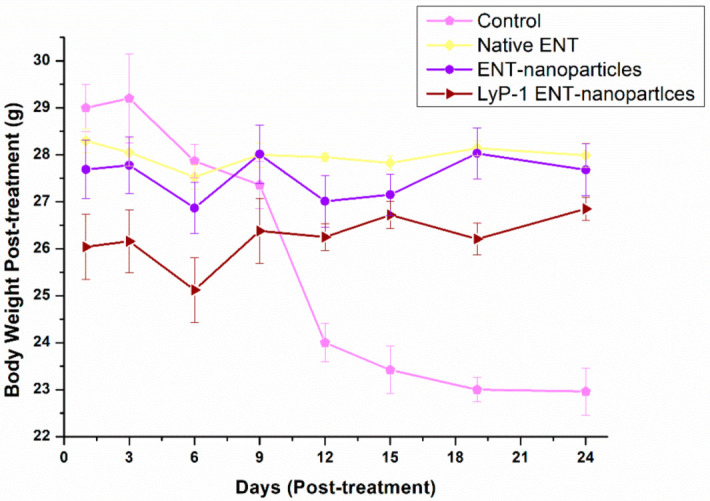
Variation in body mass of the mice over the 24-day study period. Error bars indicate ± standard deviation.

In essence, an increase in tumor size should account for a reduced total body weight due to weight loss from cancer cachexia [56,57,58]. As such, the control group with a high tumor volume displayed a reduced total body weight (29.20–22.96 g) compared with the treated group (Figure 11) (*p* < 0.05). However, there was minimal decrease in body mass recorded from mice in the treatment groups after 24 days of treatment (native ENT and the ENT-loaded LyP-1-modified nanosystem). Variation in body mass of mice occurred within the experimental groups as follows: 28.05–27.53 g (native ENT group), 27.01–28.03 g (ENT-loaded nanosystem group) and 26.72–25.12 g (ENT-loaded LyP-1-modified nanosystem group). Thus, results reveal that tumor growth was diminished by ENT and thereby decreased the loss in total body weight of the tumor-bearing mice. More importantly, the ENT-loaded LyP-1-modified nanosystem provided targeted drug delivery with superior anti-tumor effects of ENT to potentially treat OSCC.

### 2.7. Tumor Necrosis on KYSE-30 Xenograft

Tumor necrosis refers to a tumor mass having dead tissue due to inflammation, hypoxia and angiogenesis [59,60,61]. It occurs when the growth of cancer cells overwhelms the tumor blood supply. Necrosis, unlike apoptosis, is mainly responsible for potent inflammatory responses that can facilitate tumor reduction during cancer therapy [62]. Tumor necrotic factor (TNF) is often described by the loss or partial condensation of chromatin material and the absence of nuclear fragmentation, as well as compromise in membrane integrity [63].

**Figure 12:**
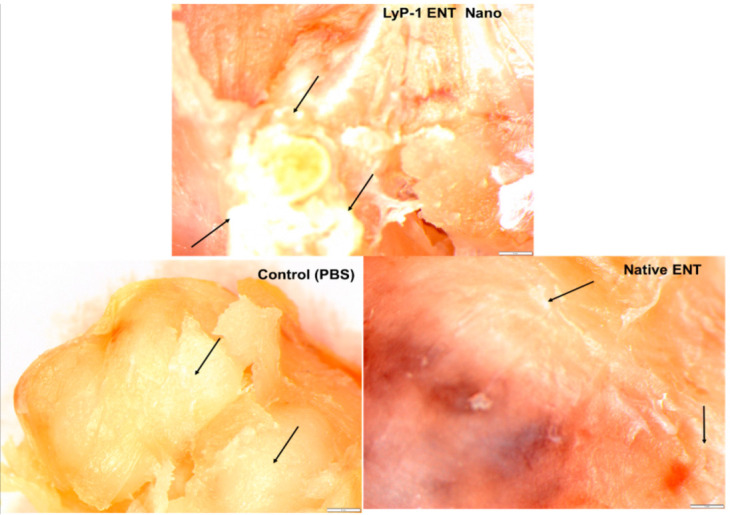
An overview of selected images of tumor necrosis after treatment with the nanosystem. Cross-sectional images were taken at high magnification (100×) using light microscopy.

As shown in Figure 12, large necrotic areas were seen within the solid tumors of mice from the treatment groups (i.e., native ENT and the ENT-loaded LyP-1-modified nanosystem) compared with the control group. Similarly, treatment aggravated the extent of tumor necrosis for the mice treated with the ENT-loaded Lyp-1-modified nanosystem relative to the native-ENT-treated tumor. These findings are in line with the results of Crescenzi and co-workers [55] who reported that L19mTNFα triggered acute anti-tumor responses in xenografts of esophageal cancer with large necrotic areas in comparison to the control tumor. The tumor necrosis observed in the control group resulted from the increased volume of the xenograft due to the rapid turn-over of esophageal tumors. In addition, as shown in Figure 7, the tumor stroma morphology appeared distorted with minimal disruption of granular-like structures and loss of the tumor ECM from experimental groups compared with the control. This finding served as additional support for the enhanced anti-angiogenic potential of ENT as a result of the more targeted form of delivery.

### 2.8. In Vivo Measurement of ENT Release from the LyP-1-Modified Nanosystem in the KYSE-30 Cell Xenograft Mice Model

**Figure 13:**
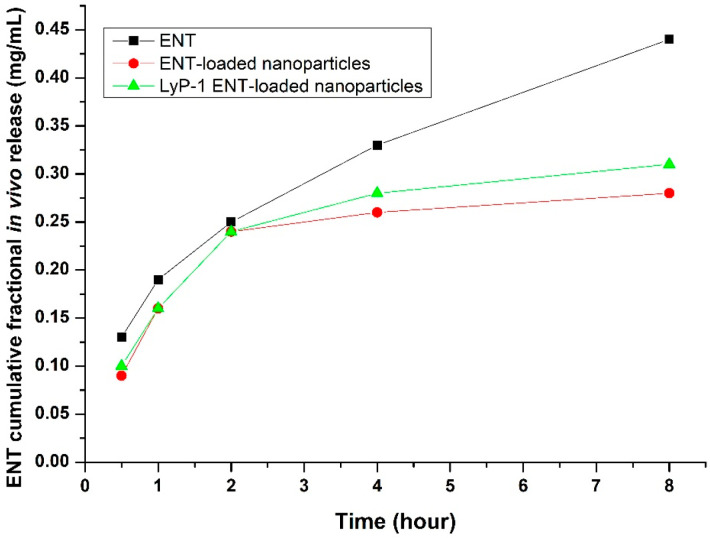
Plasma endostatin concentration profile depicting the release behavior over 8 h of native ENT relative to ENT-loaded nanoparticles, and the LyP-1-functionalized ENT-loaded nanosystem, SD = 0.021.

The in vivo release kinetics of ENT demonstrated controlled and prolonged permeation from the LyP-1-modified nanosystem into the systemic circulation. As shown in Figure 13, initial burst release values of ENT were recorded of 0.13, 0.09 and 0.10 mg/mL 30 minutes’ post-administration for pure ENT, the nanoparticles and the LyP-1-modified nanosystem, respectively. Interestingly, the ENT-loaded nanoparticles and the ENT-loaded LyP-1-modified nanosystem revealed controlled ENT release of 0.28 mg/mL and 0.31 mg/mL 8 h’ post-administration, respectively, as opposed to 0.44 mg/mL ENT released when native ENT was administered. The sustained release kinetics from the LyP-1-modified nanosystem is due to the enhanced targeting of ENT facilitated by the LyP-1 peptide on its surface causing released ENT to accumulate at the tumor site and thereby creating a drug reservoir prior to sustained diffusion into the systemic circulation [64]. Native ENT diffused rapidly into the systemic circulation with rapid clearance. Interestingly, previous studies have shown that nanoparticles increased the in vivo drug circulation time and controlled the release of the drug for enhanced therapeutic effects in relation to native drugs [65,66].

## 3. Materials and Methods

### 3.1. Materials

LyP-1 (CC (Acm) GNKRTRGC (Acm)) was purchased from Peptron, Inc. Yuseong-gu, Daejeon, Korea, having 98% HPLC purity. Branched polyethyleneimine (PEI) (MW = 25 KDa), low-molecular-weight chitosan (MW = 50 kDa, DE = 75–85%), human recombinant endostatin (MW = 22 kDa), N-hydroxysuccinimide (NHS), 1-carbonyldiimidazole (CDI), sodium tripolyphosphate (TPP) (MW = 367.86 g/mol), 1-ethyl-3-(3-dimethylaminopropyl) carbodiimide hydrochloride (EDC), poly(vinyl alcohol) (PVA) (MW = 85,000 g/mol), triethylamine dimethylformamide (DMF) and fluorescein isothiocyanate (FITC) (MW = 389.382 g/mol) were all purchased at Sigma-Aldrich (St. Louis, MO, USA) Modified NH2-PEG-COOH (Mw = 2100 g/mol) was procured from NANOCS (New York, NY, USA). Fetal bovine serum (FBS), RPMI, KYSE-30, HAM’s F12 and pentamycin/streptomycin were procured from Life Biosciences (Oakleigh, VIC, Australia) and DAPI (4′,6-diamidino-2-phenylindole) solution was obtained from Thermo Fisher Scientific (Waltham, MA, USA).

### 3.2. Methods

#### 3.2.1. Preparation of the Grafted Chitosan-Based Nanosystem

The chitosan-grafted PEI-PEG nanosystems were synthesized using a previously reported method involving the grafting of CHT to PEI [67]. A CHT solution (1.2 mg/mL) was prepared with pH adjustment to 4.97 using sodium acetate buffer (0.2 M) followed by the addition of CDI solution (50 mL, 0.000834 M). This was allowed to agitate for 60 min at 25 °C to protonate (activate) the amine group in CHT before blending with a solution of PEI (0.25% *v*/*v*; molar ratio: 2:1). Polymerization proceeded for 24 h before dialysis for a further 24 h against water using a dialysis membrane (Mw = 12,000 kD). The CHT-g-PEI conjugate was also labelled using fluorescein isothiocyanate (FITC) adapting a previously reported method [68].

#### 3.2.2. Phase 1: Conjugation of LyP-1 onto PEG-NH_2_

To synthesize the LyP-1-PEG-PEI-CHT conjugate, LyP-1 was firstly linked to the carboxylic group of the modified PEG [67]. Carboxylic activation was achieved using EDC (5 mg) and NHS (5 mg) dissolved in 0.1 M MES buffer (20 mL) under agitation for 15 min. Subsequently, LyP-1 in PBS (1.3 mg/mL; pH 7.4) was added to the activated solution and allowed to conjugate for 12 h. FITC (3.08 µmol) dissolved in DMF was added to the LyP-1-PEG-NH^2^ conjugate followed by the addition of 100 µL trimethylamine under mild agitation for 48 h at 25 °C. The resultant solution was then dialyzed against double-distilled water (DDW) for 12 h and the FITC labelled LyP-1-PEG-NH2 conjugate was stored at 4 °C until further use. The LyP-1-modified construct (LyP-1-PEG-NH2) was confirmed using Acm deprotection at Cys^2^ and Cys^10^ by employing iodine reagent [14,27].

#### 3.2.3. Phase 2: Conjugation of Synthesized LyP-1-PEG-NH_2_ onto CHT-PEI

The amino group of preformed CHT-PEI was activated using CDI chemistry for 1 h as previously reported [9]. Subsequently, the LyP-1-PEG-NH2 construct was conjugated to the amino-activated CHT-PEI solution via a terminal amino group linkage. The blend was left to react for 12 h at 4 °C and another 12 h at 25 °C to complete the polymerization process [69].

#### 3.2.4. Preparation of the ENT-Loaded Nanosystem Using the Synthesized LyP-1-PEG-NH_2_-CHT-PEI Polymeric Platform

The ENT-loaded LyP-1-functionalized nanosystem was prepared using ionotropic gelation of the synthesized conjugate. Briefly, a solution of ENT (0.5 mg/mL) was blended with a solution of FITC-labelled LyP-1-PEG-PEI-g-CHT conjugates under gentle agitation for 1 min. TPP was then added drop-wise as a polyanionic agent (0.1% *w*/*v*) and PVA (0.1% *w*/*v*) as a surfactant. The resultant ENT-loaded LyP-1-functionalized nanoparticles were centrifuged at 5000 rpm for 60 min and aggregated nanoparticles were re-suspended in DDW. Dry powdered nanoparticles were retrieved by lyophilization over 24 h and stored at −20 °C until further analysis.

#### 3.2.5. Physicochemical Property Evaluation of the LyP-1-Modified Nanosystem

Functional chemistry transformations of the LyP-1-functionalized nanosystem were determined by FTIR and NMR spectroscopy. Release of ENT from the LyP-1-functionalized nanoparticles was determined using a dialysis membrane, characterized and the particle size and zeta potential were measured by dynamic light scattering (Malvern ZetaSizer, Worcestershire, UK). TEM (FEI Tecnai T12 TEM, 60–120 kV, Hillsboro, OR, USA) and SEM (magnification: 25.00 kx) (Sigma, Zeiss Electron Microscopy, Carl Zeiss Microscopy Ltd.; Cambridge, UK) imagery facilitated the structural morphological analysis of the nanoparticles produced.

#### 3.2.6. Preparation of KYSE-30 Cell Culture

KYSE-30 cells were cultured in complete media comprising Ham’s F12 and RPMI (1:1) heat-inactivated fetal bovine serum (FBS) (10% *v*/*v*), glutamine (2 mM) and a blend of penicillin-streptomycin (100 µL) added as a supplement. The ex vivo cell study was performed under incubation with conditions constituting 5% CO_2_ at 37 °C in a humidified environment using an incubator (RS Biotech Galaxy, Irvine, UK).

#### 3.2.7. Determination of the Cytocompatibility of the Nanosystem Components and Cell Proliferation Assay

Alamar Blue (7-hydroxy-10-oxidophenoxazin-10-ium-3-one) reagent (Life Technologies, CA, USA) was used to investigate the cell cytotoxicity of the LyP-1-modified nanosystem on KYSE-30. A final concentration of 1 × 10^4^ cells/mL of KYSE-30 cells was diluted in a complete medium, seeded in a 96-well plate (25 µL/well) and incubated for 24 h before cell proliferation analysis. Serum-free culture was used to suspend different concentrations (125 µg/mL, 250 µg/mL, 500 µg/mL, 1000 µg/mL) of the native polymeric conjugates, the ENT-loaded nanosystem and the LyP-1-functionalized ENT-loaded nanosystems. Cell viability (CV %) was measured (emission/excitation; 535 nm/595 nm) after the attached cells were exposed to the nanosystems (N = 3) for 24 h using a microplate reader (FilterMax™ F5 Multi-Mode Microplate Reader, Molecular Devices, San Jose, CA, USA). Serum-free medium was used to expose the control cells.

#### 3.2.8. Assessment of Nanosystem Uptake, Internalization, Co-Localization and Sub-Cellular Biodistribution

KYSE-30 cells (1 × 10^5^ cells/well) were grown for 24 h using a previously reported method [15]. Thereafter, ENT-loaded and FITC-labelled LyP-1 PEG-PEI-g-CHT nanoparticles (2 mL at 0.1 mg/mL)) were dissolved in fresh FBS-free medium to replace the culture medium. After 24 h post-incubation at 37 °C, the medium was removed and the treated cells were rinsed with PBS solution. Finally, the nuclei of treated and control cells were stained with 10 µL/mL DAPI (4′,6-diamidino-2-phenylindole) solution and incubated for 5 min. The labelled cells were rinsed in PBS before imaging.

Cellular uptake of ENT-loaded FITC labelled LyP-1-functionalized nanoparticles was assessed using confocal microscopy. Paraformaldehyde (4%) was used to fix the attached cells in PBS for 30 min at 25 °C. Fixed cells on cover slips were treated with a solution of Triton-X100 for 5 min to instigate cell permeation. Cooled glycerol (80% *v*/*v*) was used to mount the cover slips and viewed under a confocal microscope at 495 nm (excitation) and 517 nm (emission). Similarly, the blue fluorescence of DAPI at 358 nm excitation and 461 nm emission spectra was observed.

#### 3.2.9. In Vitro Cell Migration Measurement Using Boyden Chambers

Modified Boyden chambers were used to evaluate the migration of KYSE-30 cells [67]. Briefly, a native solution of ENT-loaded conjugates (200 μL), the ENT-loaded LyP-1-functionalized nanosystem, the untreated cells and KYSE cells (6.48 × 10^4^) with serum-free media (200 μL) were transferred to the donor compartment. Medium (400 µL) containing 10% FBS was transferred to the receiver compartment as a chemo-attractant and incubated (8 h; 37 °C). Stagnant cells on the donor compartment were removed. Thereafter, using Alamar blue (1 mL), the migrated cells were measured after 4 h at 535 nm (emission) and 595 nm (excitation). Subsequently, the migrated cells were rinsed twice in PBS, fixed with 4% formaldehyde for 15 min, stained with trypan blue and viewed under a fluorescence microscope.

#### 3.2.10. Anti-Angiogenic Evaluation of VEGF and MMP2

Both VEGF and MMP2 play critical roles in tumor angiogenesis [70]. For the VEGF and MMP2 ELISA assays, each well was normalized to 774,000 cells. Aliquots were retrieved from the media at 0, 12, 24 and 48 h after incubation with the native nanosystem, the LyP-1-functionalized nanoparticles, the ENT-loaded nanoparticles and the control (serum-free media). Protein content in the cell culture supernatant was concentrated at 2000 rpm at 4 °C for 10 min using a centrifuge (Labotec, Göttingen, Germany). Protein samples were evaluated using a VEGF ELISA kit from Sigma Aldrich (Minneapolis, MN, USA) and an Abcam MMP2 Human ELISA kit with normalized protein quantities. The quantity of expressed protein was measured in duplicate at 450 nm on a microplate reader.

#### 3.2.11. Development of Tumor Xenografts and Evaluation of Anti-Necrotic Function of the ENT-Loaded LyP-1-Modified Nanosystem

Eighteen nude mice were anesthetized before subcutaneous injection of KYSE-30 cells (1 × 10^6^) in FBS-devoid culture medium was administered to the right flanks of the mice. Solid tumors emerged after 31 days post-inoculation and the diameter was measured using a digital vernier caliper. LyP-1-modified, unmodified ENT-loaded nanoparticles and placebo formulations served as treatment groups (N = 3), while PBS was administered to the control group. The treatment intervention was administered under anesthesia and a total of 1 mL of the relevant nanosystems containing an equivalent of 20 mg/kg ENT was injected subcutaneously. Equimolar subcutaneous injections of the nanosystems were administered daily (5 injections in total) of the ENT-loaded LyP-1 nanosystem (20 mg/kg), the Lyp-1-free ENT-loaded nanosystem (20 mg/kg), native ENT (4 mg/kg) and the control (PBS, pH 7.4). The mice were sacrificed and the solid tumors were harvested for further processing and analysis.

#### 3.2.12. Statistical Analysis

All assays were performed in duplicate and three independent samples were measured. Statistical significance was analyzed by using an unpaired Student’s *t*-test (Microsoft Excel) and statistical significance was expressed as *p* < 0.05. All experiments are reported as a mean (N = 3) with standard deviation (±S.D.) values where appropriate.

## 4. Conclusions

Non-specific drug targeting can be a major hurdle to the clinical efficacy of relatively new anti-cancer therapeutic molecules. The successful delivery of such drugs remains pivotal for developing chemotherapeutics with enhanced efficacy. Targeting nanosystems as described in this study, using a homing peptide viz. LyP-1 to usher ENT-loaded nanoparticles, presents a promising strategy for the site-specific delivery of the model drug ENT to achieve effective anti-angiogenic activity in SCC. The ENT-loaded LyP-1-modified nanosystem provided enhanced anti-tumor activity against KYSE-30 cells with direct targeting of tumor lymphatics, triggered nucleus rupture, mitochondrial degradation, decreased cell proliferation and migration, as well as restricted VEGF-C and MMP2 expression. In addition, a viable decrease in tumor mass and increased necrotic areas was observed in tumors after treatment. It is obvious that LyP-1 induced cell death by apoptosis. Therefore, the use of a homing peptide such as LyP-1 conjugated onto the ENT-loaded nanosystem significantly enhanced ENT release with superior in vitro and in vivo anti-tumoral efficiency for SCC over the non-targeted nanosystem.

## Data Availability

Data is contained within article.
